# Survey of information technology in Intensive Care Units in Ontario, Canada

**DOI:** 10.1186/1472-6947-8-5

**Published:** 2008-01-24

**Authors:** Stephen E Lapinsky, David Holt, David Hallett, Mohamed Abdolell, Neill KJ Adhikari

**Affiliations:** 1 Intensive Care Unit, Mount Sinai Hospital, Toronto, Canada; 2 Interdepartmental Division of Critical Care Medicine, University of Toronto, Toronto, Canada; 3 Faculty of Medicine, University of Toronto, Toronto, Canada; 4 Department of Diagnostic Radiology, Dalhousie University, Halifax, Canada; 5 Department of Critical Care Medicine, Sunnybrook Health Sciences Centre, Toronto, Canada

## Abstract

**Background:**

The Intensive Care Unit (ICU) is a data-rich environment where information technology (IT) may enhance patient care. We surveyed ICUs in the province of Ontario, Canada, to determine the availability, implementation and variability of information systems.

**Methods:**

A self-administered internet-based survey was completed by ICU directors between May and October 2006. We measured the spectrum of ICU clinical data accessible electronically, the availability of decision support tools, the availability of electronic imaging systems for radiology, the use of electronic order entry and medication administration systems, and the availability of hardware and wireless or mobile systems. We used Fisher's Exact tests to compare IT availability and Classification and Regression Trees (CART) to estimate the optimal cut-point for the number of computers per ICU bed.

**Results:**

We obtained responses from 50 hospitals (68.5% of institutions with level 3 ICUs), of which 21 (42%) were university-affiliated. The majority electronically accessed laboratory data and imaging reports (92%) and used picture archiving and communication systems (PACS) (76%). Other computing functions were less prevalent (medication administration records 46%, physician or nursing notes 26%; medication order entry 22%). No association was noted between IT availability and ICU size or university affiliation. Sites used clinical information systems from15 different vendors and 8 different PACS systems were in use. Half of the respondents described the number of computers available as insufficient. Wireless networks and mobile computing systems were used in 23 ICUs (46%).

**Conclusion:**

Ontario ICUs demontrate a high prevalence of the use of basic information technology systems. However, implementation of the more complex and potentially more beneficial applications is low. The wide variation in vendors utilized may impair information exchange, interoperability and uniform data collection.

## Background

The intensive care unit (ICU) is a data-rich environment, where information technology may enhance patient care by improving access to clinical data, reducing errors, tracking compliance with quality standards, and providing decision support [1-3]. The presence of more sophisticated information systems in the ICU has been associated with improved care [4]. Despite these potential benefits, utilization of information technology in ICUs is variable, with approximately 10–15% of U.S. ICUs in 2003 having fully implemented clinical information systems [5]. In contrast, electronic health records in other practice settings are becoming well established. For example, almost all general practices in the United Kingdom are computerized [6], as are the majority in Australia [7].

In the province of Ontario, Canada, the Ministry of Health and Long Term Care provides medical services for the province's population of 11 million through 134 acute care not-for-profit hospital corporations and an annual budget over $30 billion Canadian [8]. This single payer system offers the opportunity for standardization of information systems, but little is currently known about the implementation and availability of information technology in the province. The ICU plays a central role in the flow of patients through the health system, as the destination of transfer of the sickest patients from the emergency room and operating room. Our objective in this study was to survey ICUs across the province to identify the availability of various types of information technology and the systems and vendors utilized. We believe that this information will be essential to integrate clinical information systems into a province-wide electronic record and to identify areas for quality improvement and future research.

## Methods

### Survey development and administration

We used survey methods (item generation and reduction and clinical sensibility testing) to develop a comprehensive, self-administered internet-based survey that addressed the utilization of information technology in the ICU. We piloted the survey on 3 local intensivists. Domains of interest included:

(i) The spectrum of ICU clinical data accessible electronically (e.g. clinical, laboratory data, imaging, medications)

(ii) Availability and ease of use of computers in the ICU

(iii) Availability of decision support tools

(iv) Availability of electronic imaging systems for radiology (picture archiving and communication systems, PACS)

(v) Use of electronic order entry and medication administration systems

(vi) Use of wireless or mobile systems in the ICU

We generated an email list of Ontario ICU directors from pre-existing research and administrative email lists, as well as by a manual internet search and by contacting ICUs by telephone. Eligible ICUs were those that provided mechanical ventilation (level 3 care). We emailed an information package and link to the survey and also posted the link on a Canadian critical care listserver. The survey was carried out between May and October 2006. As an incentive, participants were offered the opportunity to enter in a draw for free registration at the Toronto Critical Care Medicine Symposium. Data were collected by a commercial internet survey application provider (Surveymonkey, Portland, OR). After the initial emailing, non-respondents received a second email, followed by a personal communication by email or telephone.

The Mount Sinai Hospital Research Ethics Board approved the study. All answers were kept confidential.

### Data analysis

Analysis was performed by hospital site for those hospitals with more than one ICU, since ICUs in the same hospital had identical information technology systems. We summarized categorical data with percentages. We used Fisher's Exact tests to analyze the association between IT availability (specifically the availability of PACS, use of an electronic Medication Administration Record and availability of computerized laboratory and imaging order entry) and university affiliation and ICU size. We carried out these statistical calculations using Statistical Analysis Software (SAS version 9.1, SAS Institute, Cary, NC). The Classification and Regression Trees (CART) method [9] was used to obtain the estimate of the optimal cut-point for the number of computers per ICU bed, and that cut-point was bootstrapped 2000 times to obtain the 95% confidence interval. We used R software (version 2.4.0) for the CART analysis [10].

## Results

We obtained responses from 50 hospitals representing 68.5% of hospitals with level 3 ICUs in the province. Of these, 21 (42%) were university-affilated and 31 (62%) used a intensivist-led management model. Twelve hospitals (24%) were in small towns (<50,000 population) and 14 (28%) in large cities (>500,000). The number of ICU beds varied, with 15 hospitals (30%) having less than 10 ICU beds, 22 hospitals (44%) having 10 to 19 beds and 13 sites (26%) with 20 or more ICU beds. The majority (64%) were medical-surgical ICUs, but various subspecialty ICUs were also represented, including trauma, cardiovascular and burns.

The majority of sites (94%) had electronic access to some component of patient clinical information, most frequently laboratory data and imaging reports (Table 1). PACS systems were available in 38 sites (76%), most of which (27 sites) reported having high definition viewing monitors available to the ICU. Few sites had the ability to capture data directly from patient monitors (7 sites, 14%) or from infusion pumps or ventilators (3 sites, 6%). Many sites reported the ability to access data remotely from elsewhere in the hospital and to a lesser extent from outside the hospital (Table 2). The most common decision support tools reported were clinical calculators, pharmacopoeias, and links to web resources (Table 3). Only 4 sites (8%) reported using electronic medication administration systems. No association was demonstrated between university affiliation or ICU size and the availability of PACS, the use of an electronic medication administration or computerized order entry (Table 4).

**Table 1:** Availability of components of the computerized clinical information system (n = 50)

**Component**	**n**	**%**
Laboratory results	46	92
Imaging reports	46	92
PACS*	38	76
Vital signs	15	30
Monitor/hemodynamic data	16	32
Medication administration record	23	46
Daily nursing notes	13	26
Daily physician notes	4	8
Order entry – labs/imaging	26	52
Order entry – medication	11	22

*PACS: picture archiving and communication systems

**Table 2:** Access to patient data outside the ICU and outside the hospital (n = 50).

Access outside the ICU, within the hospital		
**Component**	**n**	**%**

Laboratory results	49	98
PACS*	40	80
Order entry	19	38
Bedside Monitors	11	22

Access outside the hospital		

**Component**	**n**	**%**

Laboratory results	37	74
PACS*	31	62
Order entry	10	20
Bedside Monitors	4	8

*PACS: picture archiving and communication systems

**Table 3:** Availability of electronic decision support tools at sites with computerized information systems (n = 47)

**Tool**	**n**	**%**
Medical calculators	22	47
Automated management guide	6	13
Links to guidelines	18	38
Link to web resources	25	53
Diagnostic tools	8	17
Pharmacopoeia	22	47
Drug allergy alerts	21	45
Drug interaction alerts	11	23
Standardized order sets	16	34

**Table 4:** Association between university affiliation or ICU size and (i) availability of Picture Archiving and Communication Systems (PACS), (ii) electronic Medication Administration Record (eMAR) and (iii) computerized order entry, in sites with computerized systems (n = 47).

**Association with university affiliation**				
	**University affiliated % (n = 20)**	**Non-university affiliated % (n = 27)**		**p-value**

PACS	90	74		0.27
eMAR	53	48		1.0
Order entry	53	59		0.77

**Association with ICU size**				

	**<10 beds % (n = 14)**	**10–19 beds % (n = 20)**	**≥20 beds % (n = 13)**	**p-value**

PACS	79	75	92	0.52
eMAR	36	55	58	0.53
Order entry	57	47	69	0.52

The hospitals used a large variety of software vendors. Fifteen clinical information system vendors were reported, the most frequent being Meditech (Westwood, MA), GE Healthcare (Bucks, U.K.) and Cerner (Kansas City, MO). Similarly, there was no conformity in the use of PACS vendors with 8 reported, the most frequent being Agfa Impax (Mortse, Belgium) and GE Healthcare.

**Figure 1:**
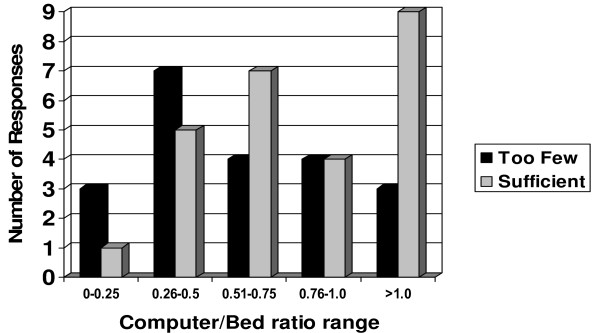
Relationship between the computer/bed ratio and physician satisfaction with the number of computers (n = 47).

Computer terminal availability in the ICU varied from as few as one computer (in small, 4 bed units) to at least one computer per bed (24% of units). Computer availability was reported as being insufficient in 21 sites (46%) (Fig 1). The perception that there are sufficient computers per bed is most differentiated by a cutpoint of 0.44 computers per bed (95% percentile confidence interval: 0.37, 1.13). Specifically, for those physicians who reported a computer to bed ratio less than 0.44, the majority, 10/12 (83%), indicated dissatisfaction. For those respondents who reported a computer to bed ratio greater or equal to 0.44, the majority, 23/37 (62%), indicated satisfaction. Multiple logins were often required to access clinical information, with the majority of sites (68%) needing 2 or more passwords. Wireless networks were installed in 23 units (46%) and mobile electronic tools were used in 28 sites (56%) (Table 5). The policy regarding use of cellular telephones varied, with 28 sites (56%) prohibiting their use throughout the hospital and 24% allowing the use of cellular telephones in some hospital areas but prohibiting use in the ICU. A policy specifying a 1 meter distance from medical devices applied to 14% of sites and 10% reported unclear or changing policies.

**Table 5:** Use of mobile electronic tools (n = 50).

**Tool**	**n**	**%**
PDA*	13	26
Tablets	2	4
Mobile computer carts	19	38
None	20	40
Unknown	2	4

*PDA, personal digital assistant (includes Palm, Pocket PC and BlackBerry devices)

## Discussion

This self-administered internet-based survey of Ontario ICUs demonstrated a high prevalence of implementation of information technology, with 92% of sites having electronic access to laboratory results and medical imaging reports and 76% using PACS systems. The use of electronic decision support systems was less prevalent and very few sites had fully capable clinical information systems or electronic medication administration systems. We found a wide variation in the use of vendors for clinical information and PACS systems, which may significantly impair information exchange and interoperability. The availability of hardware was variable, with 46% of the sites perceiving the number of computers available to be insufficient. CART analysis, using all sites including those with and without computerized provider order entry, demonstrated a cutoff between physicians satisfied with the number of computers available and those dissatisfied at a value of approximately 0.4 to 1.1 computers per bed. Approximately half the sites surveyed utilized wireless networks with mobile computing systems.

Our survey is the first inventory of information technology capacity in ICUs in a Canadian jurisdiction and provides data on a variety of information technology domains. Our response rate was typical for healthcare surveys [11]. Nonetheless, there are several limitations to this study. Common to all self-administered surveys, responses indicate self-reported rather than directly observed implementation of information technology. This methodology aimed to identify the technology available to practising physicians, but may have failed to identify existing technology that was not in common use. The information was obtained from physician-users of the systems, rather than from information technology specialists or other users, such as nurses. Nurses knowledge of available technology and requirements may be very different to physicians. We did not survey individual intensive care physicians or nurses to understand their attitudes, knowledge and behaviour regarding this information technology.

While rapid advances in computing technology are evident in commerce and industry, healthcare has lagged behind [1,2]. Information technology has the potential to improve patient safety by optimizing access to information, specifically in operations with a high information and transaction load such as drug interactions and evaluation of monitoring data [1]. The potential benefits include reduced medication errors [12], improved practitioner performance [13], and enhanced diagnostic accuracy [14]. The degree of sophistication of computing technology in the ICU has been associated with improved outcome of quality improvement initiatives to reduce catheter related bloodstream infections [4]. Although the literature demonstrating a benefit of computerized clinical decision support is growing [13,14], several studies have documented the many impediments still to be overcome [15-17]. An expectation and benefit of an ICU clinical information system is that the time that a nurse spends with documentation should be reduced, allocating more time for patient care [18,19]. However, others have demonstrated an increased documentation time following the implementation of an ICU clinical information system [20]. Furthermore, new software systems can actually facilitate rather than reduce some types of error [21]. Appropriate assessment of any new technology prior to implementation remains essential [22].

An often quoted barrier to implementation of clinical information systems is the required change in culture to healthcare workers [23,24]. While this may be true in other clinical areas, our study demonstrates that computing systems are currently an integral component of the ICU. Having overcome these initial barriers, the next step is to introduce the more sophisticated and potentially more beneficial components of the clinical information system. The ICU is a data-rich environment at risk for data overload, and there may be significant benefit to safety and quality of care from decision support applications, medication administration systems and computerized order entry [24]. Our data confirm the lack of standardization of software across the province, with 15 vendors of clinical information systems being used. As new systems are implemented or updated, it is essential that standardization be addressed, to allow data transfer between systems and to reduce the potential for errors related to inadequate familiarity with the software [21].

## Conclusion

Providing efficient, safe, individualized care in the data-rich environment of the ICU can only be achieved with the use of information technology. We have demonstrated a significant level of early implementation in Ontario ICUs but further investment is needed. The variation in systems in use is concerning, and standardization and interoperability need to be addressed.

## Key messages

• Almost all ICUs in the province of Ontario, Canada, have electronic access to some component of patient information, most frequently laboratory data and imaging.

• In contrast, use of decision support systems, electronic medication administration systems and full clinical information systems is very uncommon.

• Multiple different IT vendors are used, which may impair information exchange and interoperability.

## List of abbreviations

IT, information technology

PACS, picture archiving and communication systems

eMAR, electronic medication administration record

## Competing interests

The author(s) declare that they have no competing interests.

## Authors' contributions

SL contributed to the design of the study, data collection and analysis, and drafted the initial version of the article. DH was responsible for the design of the study, data analysis, and contributed to drafting the article. DH contributed to the data collection and was responsible for the statistical analysis. MA contributed to the data analysis and to drafting the article. NA contributed to the design of the study, data analysis, and critically revised the manuscript. All authors read and approved the final manuscript.

## Pre-publication history

The pre-publication history for this paper can be accessed here:


